# Echoes from northern Iberia: distribution, ecology, genetics, and identification of Asturian cicadas (Hemiptera: Cicadidae)

**DOI:** 10.1093/jisesa/ieag065

**Published:** 2026-06-30

**Authors:** Jairo Robla, Vera L Nunes, Iván Orois, Martiño Cabana, Jorge Rodríguez-Pérez, Omar Sánchez, Saúl R Rodríguez

**Affiliations:** Biological Collections, Doñana Biological Station (EBD-CSIC), Sevilla, Spain; Centre for Ecology, Evolution and Environmental Changes & CHANGE-Global Change and Sustainability Institute, Faculdade de Ciências da Universidade de Lisboa, Lisbon, Portugal; Independent Researcher, Arzúa, A Coruña, Spain; Grupo de Investigación en Bioloxía Evolutiva (GIBE), Departamento de Bioloxía, Facultade de Ciencias, Universidade da Coruña. Campus da Zapateira, Coruña, Spain; Biodiversity Research Institute, CSIC—University of Oviedo—Principality of Asturias, Mieres, Spain; Department of Biology of Organisms and Systems (Zoology), University of Oviedo, Oviedo, Spain; Independent Researcher, Gijón, Principado de Asturias, Spain

**Keywords:** distribution modeling, identification key, phylogeny, species ecology, sonograms

## Abstract

Cicadas are a group of insects recognized for their distinctive life cycle and the stridulatory calls produced by males to attract females. While cicadas are commonly associated with thermophilic forests and xerophilic shrublands in Mediterranean regions, their distribution in temperate areas of Europe remains poorly understood. This study focuses on the cicadas of the northern Iberian Peninsula, specifically Asturias, characterized by a temperate climate. Over three years of fieldwork, we surveyed potential sites to locate cicada populations. Efforts were made to collect data on their habitat, ecology, and behavior. Acoustic recordings were used to characterize species-specific calling songs, and specimens were captured for genetic analyses. In addition, species distribution models were developed using climatic variables to assess potential habitat suitability across the region. We identified four cicada taxa—*Cicada orni*, *Tibicina quadrisignata*, *Tettigettalna argentata*, and *Cicadetta* sp.—primarily occupying forested habitats, native shrublands, and pine plantations. These species were found between 200 and 1,600 m a.s.l. and were acoustically active at temperatures above 18 to 21 °C, preferentially during sunny and low-wind conditions. Distribution models highlighted the warmest valleys of the study area as the most favorable for cicada occurrence, providing guidance for future sampling efforts. Furthermore, we provide the first genetic characterization of the cicada species detected in the region, which supports the morphological and acoustic identifications and reveals the presence of a putative new species of the genus *Cicadetta*. Finally, we outline conservation-relevant considerations for the preservation of cicada populations in temperate environments.

## Introduction

Cicadas (Hemiptera: Cicadidae) are insects widely distributed worldwide, mainly across temperate and tropical regions (Marshall et al. 2018). One of their most distinctive traits is their unusually long-life cycle (Boulard and Mondon 1995, Williams and Simon 1995). The subterranean juvenile stage is the most prolonged, during which individuals feed on the xylem sap they extract from the roots of their host plants (Boulard and Mondon 1995). Emergence usually takes place in summer, when the nymphs leave behind their exuviae and transform into winged, epigeous adults. These adults feed on nutrients they extract from plant stems and branches (Boulard and Mondon 1995). Their best-known feature is the characteristic “song” that males use to attract females for mating (Boulard 2006, Marshall et al. 2018), which is often species-specific and therefore an important taxonomical character (Sueur 2006, Pinto-Juma et al. 2009). This song is produced by the timbal, a specialized organ that may rapidly contract and relax by the action of strong muscles, causing the hardened timbal plates to buckle in and out and resonate within the hollow abdomen, a mechanism that has long been recognized in cicada bioacoustics (Pierce 1948, Boulard 1977, Bennet-Clark and Young 1992, Fonseca 2013).

Regarding their ecology, these species are abundant in forests and grasslands (Koenig and Liebhold 2005), and they also occupy many typical xerophilic shrubland habitats worldwide (Puissant and Sueur 2010, Sanborn 2013, Sanborn and Phillips 2013, Stephen 2021), as well as certain urban environments (Nguyen et al. 2018, Pons et al. 2021). Cicada species may adapt to occupy different vegetation strata, from herbaceous and shrubby to arboreal, where they sing on warmer days and where the host plants they feed on are found (Quartau et al. 2004, Puissant and Sueur 2010, Sanborn et al. 2011). Most species are thermophilic, selecting warm habitats and increasing the number of syllables per second in their songs as ambient temperature rises (Fonseca and Allen Revez 2002, Sanborn et al. 2011). Although some species have been considered important as crop pests in some parts of the world (Mehdipour et al. 2016, del Castillo et al. 2025, de Souza Covre and Flechtmann 2025, de Souza et al. 2025), most cicadas have negligible consequences on crop yields.

In Europe, the Cicadidae have been the focus of many studies, with several new species and subspecies described in recent years, reflecting the cryptic biodiversity of this group (eg Gogala et al. 2008, 2009, 2011, Puissant and Sueur 2010, Hertach 2011, 2021, Simões et al. 2013, Hertach et al. 2015). The family is represented in Europe by three subfamilies (see Marshall et al. 2018, Gogala 2025). First, Cicadinae includes two genera (*Cicada* Linnaeus, 1758; and *Lyristes* Horváth, 1926) and comprises some of the largest and most widespread cicadas, often found in urban environments (Pons et al. 2021). Second, Cicadettinae, is the most diverse subfamily with up to 13 genera: *Cicadatra* Kolenati, 1857; *Cicadetta* Kolenati, 1857; Dimissalna Boulard, 2007; *Euboeana*  Gogala et al. 2011; *Euryphara* Horváth, 1912; Hilaphura Webb, 1979; *Oligoglena* Horváth, 1912; *Pagiphora* Horváth, 1912; Pseudotettigetta Puissant, 2010; *Tympanistalna*  Boulard 1982; Tettigettalna Puissant, 2010; Tettigettula Puissant, 2010; and Tettigettacula Puissant, 2010 (Marshall et al. 2018, Gogala 2025). This subfamily has been frequently under taxonomic review in Europe, with the description of several new taxa and reclassifications published in recent years (eg Puissant and Sueur 2010, Hertach 2011, 2021, Hertach et al. 2015). Finally, Tibicininae is represented only by the genus *Tibicina* Kolenati, 1857 (Gogala 2025). Current knowledge of species diversity in Europe and male calling songs is periodically updated at www.cicadasong.eu (Gogala 2025). However, the distribution, genetic data, ecology, and even the life cycle of several species remain poorly known and require further study.

In the Iberian Peninsula, studies on Cicadidae have often been sporadic and patchy, typically focusing on regions harboring the highest endemicity (mainly Catalonia, Andalusia, and Portugal) and on a few taxa showing cryptic diversity (eg *Cicada* and *Tettigettalna*) (see Gogala 2025). Since the first comprehensive Cicadidae catalogue for Spain (Gómez-Menor Ortega 1957) and for Portugal (Boulard 1982) were published, some improvement was provided by scattered studies over the last 40 years (Boulard 2000, Quartau et al. 2004, Aguin-Pombo et al. 2007, 2008, Puissant and Sueur 2010, Hertach et al. 2016, Pons et al. 2021, Mora-Rubio 2022). Currently, there are a total of 25 cicada species with confirmed occurrence in the Iberian Peninsula according to existing literature. Cicadettinae are the most diverse, with 18 species, 14 of these being considered endemic to the territory. However, central and northern regions have received little attention, with the temperate northern peninsula remaining entirely unstudied during this period. This knowledge gap may reflect a historical bias in survey effort, as these regions have generally been considered less suitable for cicadas because of their milder summers.

Since the geographic position and topography of the Iberian Peninsula promote the coexistence of two climates, from temperate zones in the north to Mediterranean climates in the center and south, the northern areas turn into interesting zones to study the occurrence of cicadas adapted to milder climates and their response to a warming climate. We present the first data on the cicadas of the Cantabrian region and the temperate northern Iberian Peninsula, with a focus on Asturias. Specifically, the aims of this work are: (a) to document the known and potential distribution of cicada species in Asturias, (b) to summarize on species occurring in closest areas, (c) to comment information on the biology, ecology, habitat, and behavior of the recorded taxa, (d) to provide visual and acoustic identification keys for Asturian cicadas for untrained nature enthusiasts and citizen science boosting, (e) to generate novel genetic data for all species detected in the study area, and (f) to discuss conservation-relevant aspects in order to guide and support future studies on northern Iberian cicada faunas.

## Materials and Methods

### Study Area

We focused sampling in the Principality of Asturias (hereafter, Asturias) in northern Spain. Located along the Cantabrian coast, this region is characterized by a temperate oceanic climate (MARM 2011). The mean annual temperature ranges between 12 and 14 °C, with mild winters and moderately (to low) warm summers (MARM 2011). Precipitation is relatively high, averaging 1,000 to 1,500 mm per year, and is distributed evenly throughout the year (MARM 2011). Sunshine hours are limited compared to other regions of Spain due to the frequent cloud cover (MARM 2011). This region exhibits a wide range of habitats across its altitudinal gradient (from sea level to 2,650 m), ranging from coastal areas and riparian forests at low elevations, to deciduous woodlands and pastures at mid-elevations, and high-mountain shrublands and grasslands at higher altitudes (MITECO 2003). Human-influenced landscapes, including agricultural fields and urban areas, are also present, highlighting the ecological diversity of the region (MITECO 2003).

### Field Sampling and Audio Processing

Sampling was conducted in Asturias between May 2023 and August 2025, spanning three consecutive seasons. Active sampling months were June through September, when adults were observed flying and singing. The first cicada records included in this study were obtained opportunistically during unrelated fieldwork conducted by the authors. In subsequent seasons, areas surrounding these initial records were intensively surveyed. Sampling consisted of nonsystematic transects along roads and pathways, aimed at detecting the presence of calling males. The transect design was oriented toward maximizing the number of UTM 10 × 10 km and 5 × 5 km grid cells with confirmed presence. Once a locality with cicada activity was identified, a series of standardized procedures was followed. First, accurate (up to 10 m) GPS location data was registered via the Epicollect5 Smartphone app. Then, the general vegetation at each site was characterized, noting both arboreal and shrubby species. Third, and after locating and preliminarily identifying cicadas by their acoustic signals, we recorded the calling song of some males with a Zoom H1n recorder (sometimes with a Birdmic parabolic microphone) positioned near the individual (2 to 5 m) for up to five minutes to obtain representative sonograms. When the recorder was unavailable, we used One Plus 12 and Poco X3 Pro smartphones under the same protocol, but only for species confirmation, not for analysis, due to the technical limitations of these recording devices. Whenever possible, in situ behavioral observations were also made, annotating any data relevant to the biology and ecology of the species. These records included the host plant species on which cicadas fed; perch position on the host plant during singing; flight and courtship behaviors; potential predators; and the ambient temperature and time of day at which singing occurred. Finally, to confirm identifications and prepare reference material for entomological collections, selected specimens were captured using extendable insect nets. Captured individuals were euthanized by freezing and subsequently mounted with entomological pins. All specimens are currently held in the collection of the first author, and several specimens will be deposited in the reference entomological collections of Spain.

Software Audacity 3.5.1 and Raven Lite 2.0.5 were used to analyze the recorded calling songs. Oscillograms, sonograms, and associated statistics (eg peak, maximum, and minimum frequency) were obtained from polished recordings with the *Rthoptera* package (Rivas et al. 2025) in R Studio 4.2.2. Distribution maps were generated with QGIS 3.30.3, depicting 5 × 5 km UTM grid cells (EPGS: 25829-ETRS89) with at least one recorded occurrence of the target species. Specimen and habitat photographs were taken with Canon EOS 90d and Canon EF-S 18-135mm f/3.5-5.6 IS USM lens and processed with Pixelmator pro 3.7.

### Genetic Analysis

Selected specimens of different cicada species found were subjected to the DNA extraction process to confirm the previous morphological identifications. The DNA was extracted from 30 mg of ethanol-preserved tissues from one leg, using the E.Z.N.A. Tissue DNA Kit (Omega Bio-tek). DNA samples were stored at −20 °C. The mitochondrial cytochrome c-oxidase subunit I (COI) and subunit II (COII) gene fragments were amplified by Polymerase Chain Reaction (PCR) in a total volume of 30 μl, using the following primer pairs: (1) LCO1490/HCO2198 (Folmer et al. 1994) for COI-5P; (2) C1-J-2195/TL2-N-3014 (Simon et al. 1994) for COI-3P, and (3) TL2-J-3034/TKN-3786 (Simon et al. 1994) for COII. The reaction mixture contained 2.5 μl template DNA, 2.5 μl of 25 mM MgCl_2_, 4 μl of 2.5 mM dNTPs, 1 μl of 10 μM primers, 0.15 μl Taq polymerase (GoTaq G2 Flexi DNA Polymerase 5U⁄μl), and 8 μl of 5 × Buffer GoTaq Promega (1× final concentration). PCR conditions consisted of an initial denaturation step of 95 °C for 4 min, followed by 35 amplification cycles (95 °C for 45 s, 48 °C for 45 s, 72 °C for 30 s), and a final elongation step at 72 °C for 7 min. Successful amplification of gene fragments was checked with horizontal electrophoresis (1% agarose gel) with 0.05 μl/ml of Simply Safe (EURx Ltd. 80-297 Gdańsk, Poland). PCR products were lately purified with Agarose-Out DNA Purification Kit (EURx Ltd. 80-297 Gdańsk, Poland). Finally, forward and reverse sequencing was performed at MACROGEN (Madrid, Spain), using the standard Sanger sequencing method (Sanger and Coulson 1975).

The forward and reverse sequences obtained by Sanger sequencing were edited using Geneious Prime v2025.0.3 (https://www.geneious.com) for quality trimming and primer removal. Then, they were aligned and manually checked to correct any possible wrong base calling. A genetic species identification was attempted using nBlast implemented in Geneious Prime using the default values to search in GenBank databases. For constructing the phylogenetic trees, the tree novel sequences of *Cicadetta* sp. from Asturias were aligned with a set of 94 previously published sequences deposited in GenBank of different European *Cicadetta* species. The list of accession numbers can be checked in Supplementary Appendix S1. Phylogenetic analyses were conducted using IQ-TREE v3.0.1software (Minh et al. 2020). The Model Finder option included in IQ-TREE was used to predict the nucleotide substitution model showing the best BIC and AICc scores (Kalyaanamoorthy et al. 2017). A Maximum Likelihood tree was performed using Ultrafast Bootstrap options (1000 bootstrap replicates) (Minh et al. 2013), and a search was conducted for the best-scoring tree using the HKY + F + I model for COI-3P and COII fragments individually.

### Potential Distribution Models

The ecological niche models were developed in accordance with the recommendations provided by Sillero et al. (2021). We used the 19 bioclimatic variables from WorldClim v2 (Fick and Hijmans 2017), at a resolution of 30 arc-seconds (Supplementary Material S1). Additionally, we included a 30-m resolution digital elevation model (DEM) from EarthData (https://search.earthdata.nasa.gov/), from which we calculated slope using the *terra* package in R (Hijmans 2024). We then resampled the 21 variables to the extent of the Principality of Asturias and at a resolution of 1 km (UTM zone 29 T, ETRS89 datum). Highly correlated variables (Pearson correlation coefficient > 0.75; Dormann et al. 2013) were excluded, selecting the most biologically relevant variable from each pair of correlated variables. Finally, we also performed a Variance Inflation Factor (VIF) analysis, excluding variables with VIF > 10 (Dormann et al. 2013). Our final dataset contained six variables: mean diurnal range (Bio2), isothermality (Bio3), maximum temperature of warmest month (Bio5), annual precipitation (Bio12), precipitation of driest quarter (Bio17), and slope. Models were built using Generalized Linear Model (GLM), Generalized Boosting Model (ie boosted regression trees; GBM), Generalized Additive Model (GAM), Random Forest (RF), Multiple Adaptive Regression Splines (MARS), and maximum entropy (MaxNet) algorithms, implemented through the *biomod2* package (Guéguen et al. 2025). Ten thousand random pseudo-absences were generated. Each model was replicated three times, using 70% of the data for training and 30% for validation. Model performance was evaluated using AUC (Area Under the ROC Curve) and TSS (True Skill Statistic). To generate a consensus prediction of species distribution, we applied an ensemble modeling approach following Araújo and New (2007). This method combines the outputs of multiple individual models to reduce uncertainty and improve predictive performance. Only those models with an AUC score > 0.8 were retained for inclusion in the ensemble. Final models were projected across Asturias to produce continuous environmental suitability maps.

## Results

### List of Detected Species

We sampled 25 10 × 10 UTM squares, representing approximately 25% of Asturias (10,600 km^2^). We recorded four distinct species in Asturias (all of them new for the region), representing all three European subfamilies: *Cicada orni* L., 1758, *Tibicina quadrisignata* (Hagen, 1855), *Tettigettalna argentata* (Olivier, 1790), and *Cicadetta* sp. Kolenati, 1857. The most frequently detected taxon was *Te. argentata* with a total of 170 records, followed by *Ti. quadrisignata* with 45, *Cicadetta* sp. with 27, and *C. orni* with 10. A summary of potential species for Asturias could be seen in Supplementary Appendix S2.

#### Subfamily Cicadinae Batsch, 1789

***Cicada orni*** L., 1758

See Fig. 1A–D

**Fig. 1.**
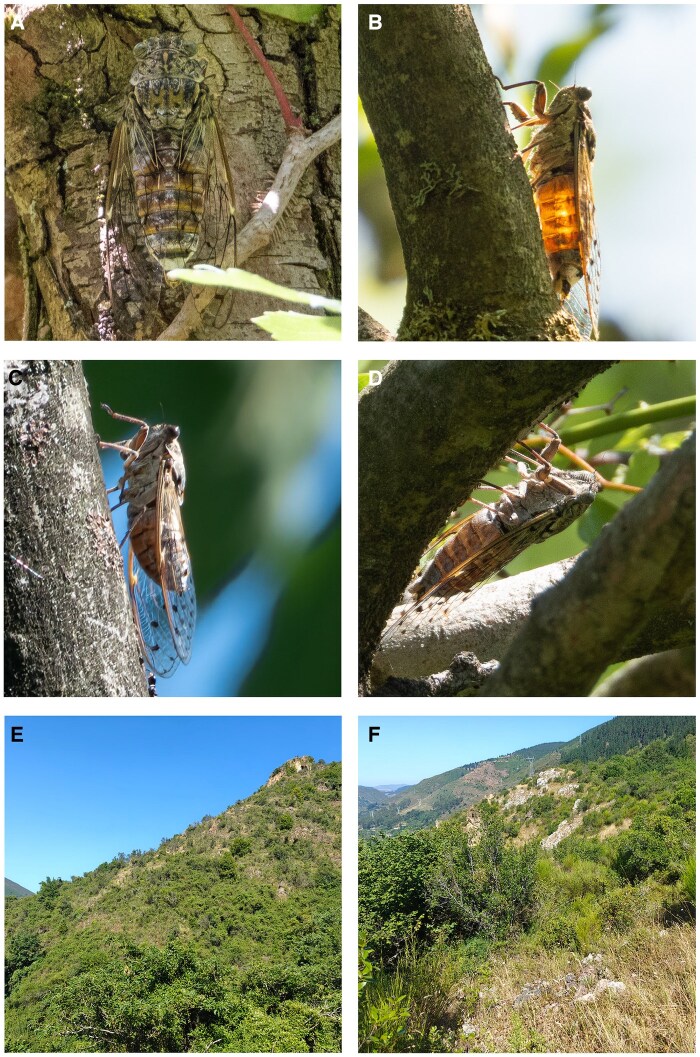
Alive specimens of *C. orni* in different plants (A–D); in Castiello (Tineo). Habitats of *Cicada orni* in (E) narrow-leaved ash *Fraxinus angustifolia*, chestnut *Castanea sativa* in Castiello (Tineo), and (F) hawthorn *Crataegus monogyna* in Castiello (Tineo).

**Fig. 2.**
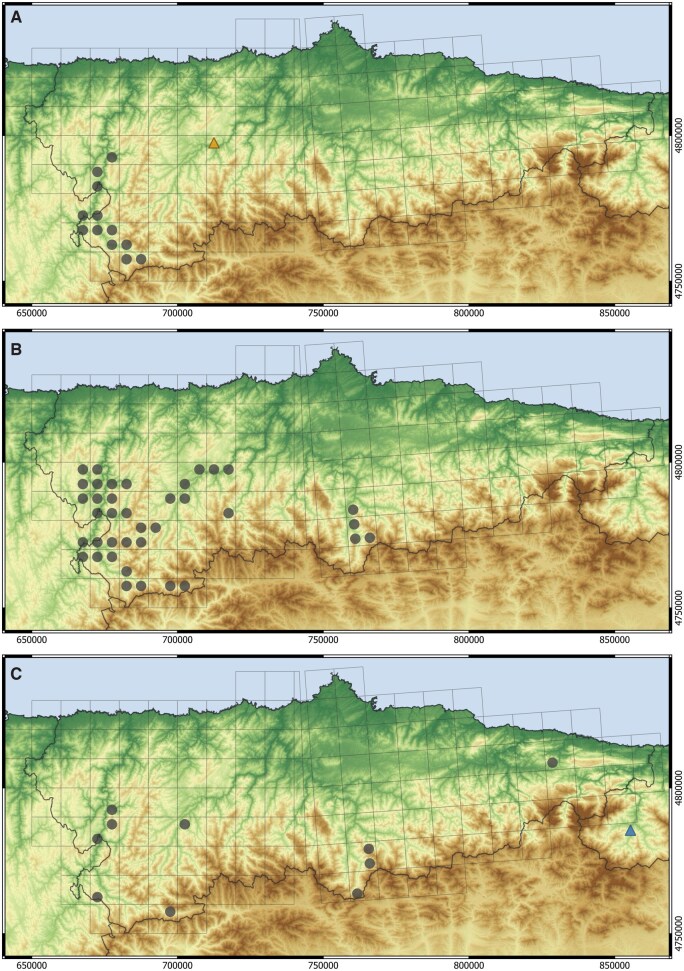
Distribution of Cicadidae in Asturias in 5 × 5 km UTM grid cells. (A) *Cicada orni* (orange triangle) and *Tibicina quadrisignata* (black dots); (B) *Tettigettalna argentata*; (C) *Cicadetta* sp. (black dots) and *Lyristes plebejus* (blue triangle). Grey grid represents 10 × 10 km UTM squares.

*Distribution and abundance.* This species has only been recorded in a single 10 × 10 km UTM grid in western Asturias (Fig. 2A), in a particularly thermophilous area (only in the middle basin of the Narcea River), where it is locally abundant. A list of all records with more precise data can be found in Supplementary Material S2.

*Habitat. Cicada orni* was found in a riparian valley in different habitats. In one of them, the vegetation cover was patchy but mostly dense, composed mainly of thermophilous deciduous forest, with dominant species like chestnut (*Castanea sativa* Mill), pedunculated oak *Quercus robur* L., narrow-leaved ash (*Fraxinus angustifolia* Vahl), and sycamore (*Acer pseudoplatanus* L) (Fig. 1E and F). The species is also present in areas of shrubland with Mediterranean buckthorn (*Rhamnus alaternus* L.), hawthorn (*Crataegus monogyna* Jacq.), laurel (*Laurus nobilis* L.), and sloe (*Prunus spinosa* L.) bushes growing on limestone soils. They also occupied meadows interspersed with mixed deciduous forest with beech (*Fagus sylvatica* L.), hazel (*Corylus avellana* L.), holly oak (*Quercus rotundifolia* Lam.), and oaks and chestnuts (Fig. 1E and F). The only known population occurs near water sources, with all contacts less than 400 m a.s.l. away from the La Barca reservoir in the Narcea River. Its altitudinal range spans from 216 to 362 m a.s.l.

**Fig. 3.**
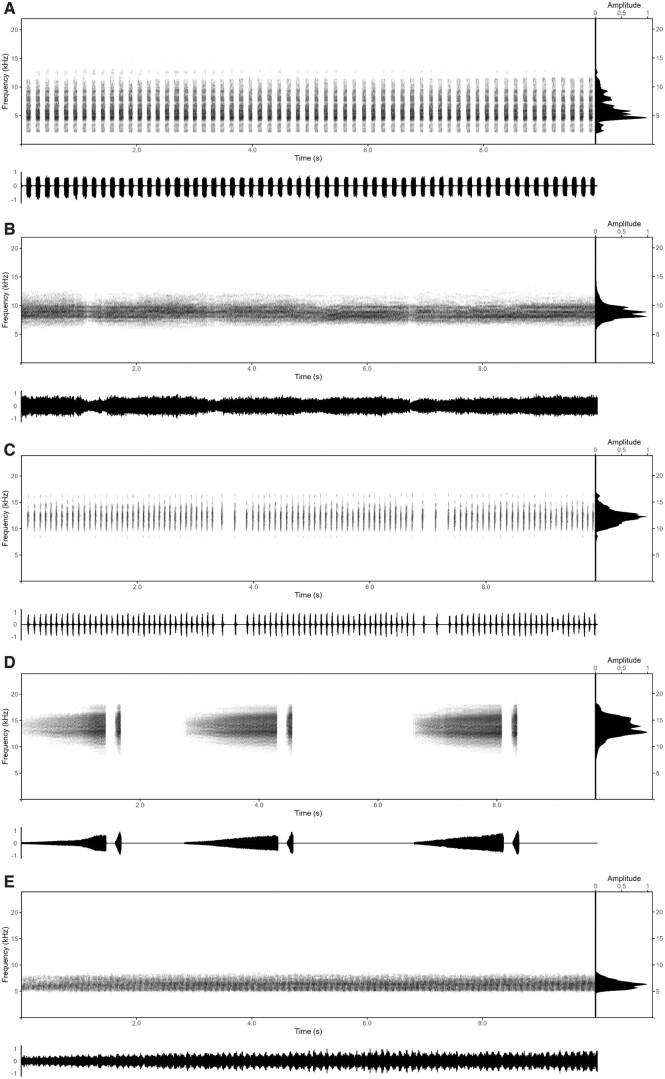
Acoustic characteristics of Asturian Cicadidae represented via Multiplots. Each plot represents a spectrogram (main plot), an oscillogram (bottom plot), and a power spectrum (left plot). Espectrograms represent variation in frequency, while oscillograms describe changes in amplitude (energy). Power spectrums portray the cumulative amplitude across frequencies. Represented species are: (A) *Cicada orni*, (B) *Tibicina quadrisignata*, (C) *Tettigettalna argentata*, (D) *Cicadetta* sp. and (E) *Lyristes plebejus*.

*Ecology and acoustic behavior.* This species selected narrow-leaved ash (*Fraxinus angustifolia*), pedunculated oak (*Quercus robur*), and sycamore (*Acer pseudoplatanus*) to sing. They select the top parts of surrounding vegetation (from 5 m in *C. monogyna* to 20 m in *F. angustifolia*), preferably in forested areas instead of shrubland. Male *C. orni* started singing by mid-day until late-afternoon with a synergic song of all individuals in the area, when temperatures rose above 25 to 28 °C. They were seen feeding on *Q. robur*. Specimens of this species were only detected in the month of July, although the same spots were visited in other months. The recorded calling song of this species (Fig. 3A) corresponds to a monotonous and regular pattern with a rate of 5 to 6 schemes per second and 4.8 kHz of peak frequency that matches the typical song of the species as reported in Pinto-Juma et al. (2005).

*Molecular identification.* Identification of the new sequence of the Asturian *C. orni* specimen (COI-5P; PX467326) with the NCBI BLAST algorithm returned 99.66% to 99.85% pairwise identity with three sequences of *C. orni.* The remaining hits belong to other species of the genera *Cicada*, *Psithyristria*, and *Meimuna*, with a similarity of 95.04% or less.

#### Subfamily Tibicininae Distant, 1905

**Fig. 4.**
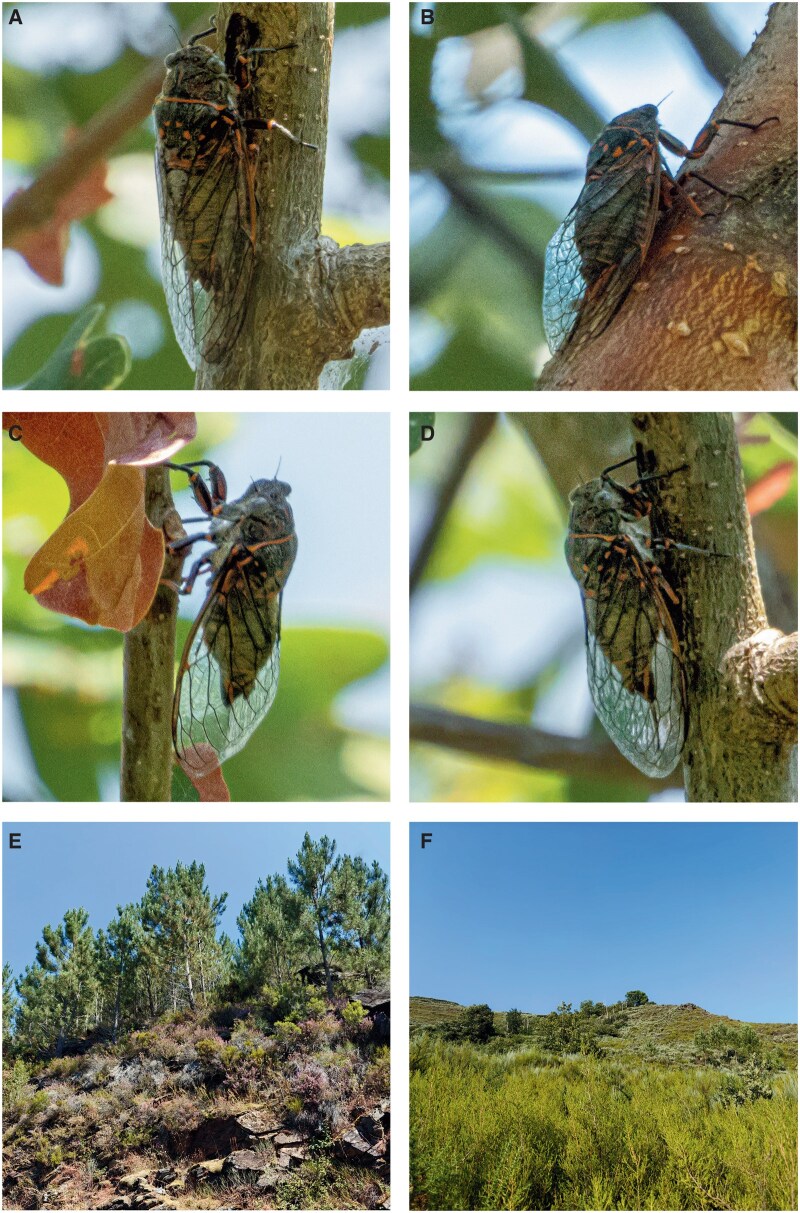
Alive specimens of *Tibicina quadrisignata* in different plants (A–D), in Tresmonte da Buliqueira (Grandas de Salime). Habitats of *Ti. quadrisignata* in (E) pine plantations of *Pinus* spp., showing a preference for sunny pine woodland, heathland habitats with *Erica*, *Cytisus*, and other shrub species, mixed with strawberry trees *Arbutus unedo* in Murias (Allande) and (F) pedunculate oak *Quercus robur and* pyrenean oak *Quercus pyrenaica* heathland habitats with *Erica*, *Cytisus*, and other shrub species in Tresmonte da Buliqueira (Grandas de Salime).

***Tibicina quadrisignata*** (Hagen, 1855) See Fig. 4A–D

*Distribution and abundance.* This species has been recorded with a distribution concentrated in the south-western part of Asturias, close to the border with Galicia (Fig. 2A), restricted to the warmest valleys (Navia-Oro and Ibias-Aviouga rivers), being abundant only in some localities. A list of all records with more precise data can be found in Supplementary Material S2.

*Habitat.* This species occurs in river canyons, particularly in south-facing slopes. It is found in dense forests with pedunculate oak (*Quercus robur*) or cork oak (*Quercus suber* L.), and in pine plantations of *Pinus* L. spp., showing a preference for sunny pine Woodland (Fig. 4E and F). It also selects heathland habitats with *Erica* L., *Cytisus* Desf. and other shrub species, mixed with strawberry trees (*Arbutus unedo* L.) (Fig. 4E and F), even close to burned areas. Its altitudinal range spans from 277 to 734 m a.s.l.

*Ecology and acoustic behavior.* Individuals of *Ti. quadrisignata* are elusive, selecting densely vegetated areas to sing. The species tends to choose medium-upper portions of trees or shrubs, where males often perch on the tips of branches. Individuals are shy and often fly 100- to 500-m distances if disturbed. Records of the species were obtained between mid-July and mid-August. It must be noted that the song pattern and frequency are nearly identical to *Ti. garricola*, another species also occurring in Iberia. The song sounds like a long and monotonous buzz that is higher-pitched than the song of *C. orni* (7 to 10 kHz) (Fig. 3B).

*Molecular identification.* Identification of the sequence of the Asturian *Tibicina* specimen (COI-3P; PX467325) with the NCBI BLAST algorithm resulted in 98.40% pairwise identity with the only sequence of *Ti. quadrisignata* available in the database. The remaining hits were with other species of the genus *Tibicina* and *Okanagana*, with a similarity of 92.81% or less. Sequences for the COI-5P fragment for the *Tibicina* genus were not available in public databases.

#### Subfamily Cicadettinae Buckton, 1890

**Fig. 5.**
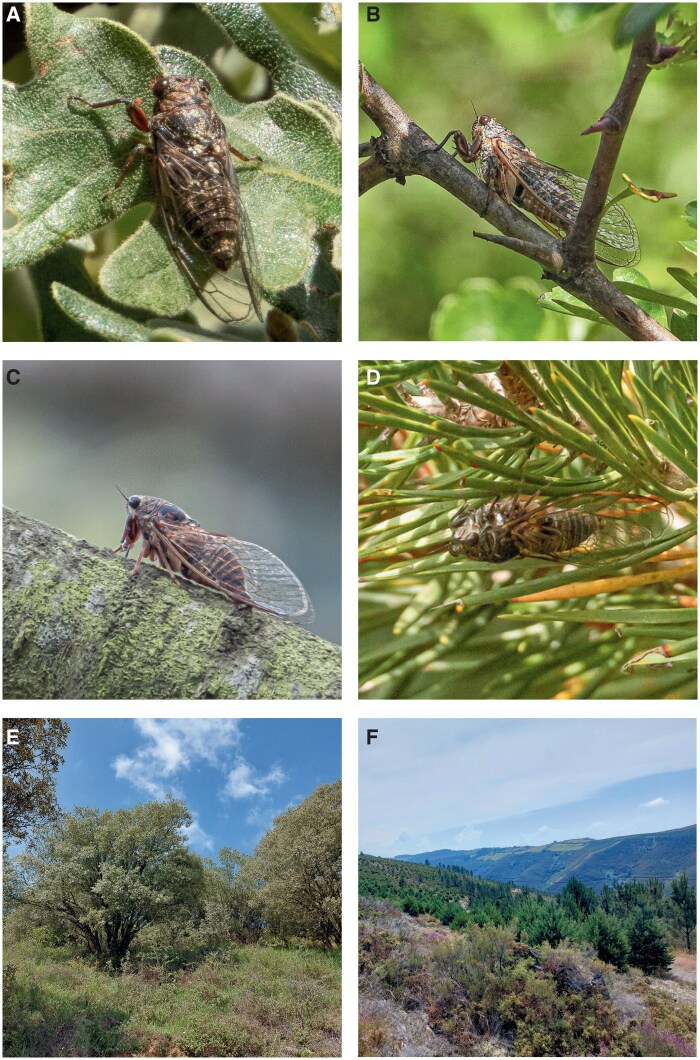
Alive specimens of *Tettigettalna argentata* in different plants (A–D). Habitats of *Te. argentata* in (E) holly oak *Quercus rotundifolia* in Columbiello (Lena) and (F) in pine plantations of *Pinus* spp. in Monte dos Vilares (Ibias).

***Tettigettalna argentata*** (Olivier, 1790) See Fig. 5A–D

*Distribution and abundance.* This species has been recorded with a scattered distribution in three areas of Asturias: the south-western zone, the eastern zone, and also appeared in southern Asturias (Fig. 2B), namely across all thermal valleys (Navia-Oro, Ibias-Aviouga, and Narcea-Coto rivers), as well as the Lena-Pajares rivers. This is the species more easily detected and more extensively distributed in the study area, being very abundant throughout its area of occurrence. A list of all records with more precise data can be found in Supplementary Material S2.

*Habitat.* This species is the most widely recorded across the territory, occupying a variety of habitats. It can be found in forests mainly composed of *Q. rotundifolia* and deciduous oak species like *Q. robur* and *Q. pyrenaica* Willd. (Fig. 5E and F). *Tettigettalna argentata also* occupied low shrublands with *Erica*, *Cytisus*, and *Genista* L.; pine plantations of *Pinus sylvestris* L. or *Pinus pinaste*r Aiton, and riverside forests with Iberian alder (*Alnus lusitanica* Vít, Douda & Mandák) and *Corylus avellana* (Fig. 5E and F). They can be found along road margins within roadside vegetation and seem to persist well on recently burned areas. *Tettigettalna argentata* is the only species of cicada present in Asturias that inhabits *Eucalyptus* L’Hér. plantations, although they are not a preferred habitat. Its range spans from 200 to 900 m a.s.l.

*Ecology and acoustic behavior. Tettigettalna argentata* males sing indistinctively from high or low vegetation and can be active at temperatures as low as 20 to 21 °C. Females often perch on low vegetation near singing males, frequently moving between listening spots with short and erratic flights. Males of this species often sing from more exposed areas than other cicada species. There are recorded instances of the species feeding on top branches of holly oak *Quercus rotundifolia. Te. argentata* has a longer period of activity than other species in Asturias, as records of the species were obtained from June to mid-late August. The song pattern is composed of the repetition of long trains of short and fast-paced chirps (roughly 30 chirps or more) of 10 to 15 kHz (Fig. 3C). One of the captured specimens had an unidentified mite (Acari) attached to the abdomen of the cicada.

*Molecular identification.* The Blast identification engine identified the new sequence of *Te. argentata* specimen (COI-5P; PX467329) with 96.90% to 99.09% pairwise identity with the available sequences of *Te. argentata*. The remaining hits were with other species of *Tettigettalna* with a similarity of 93.01% or less.

#### *Cicadetta* sp.

See Fig. 6A–D

**Fig. 6.**
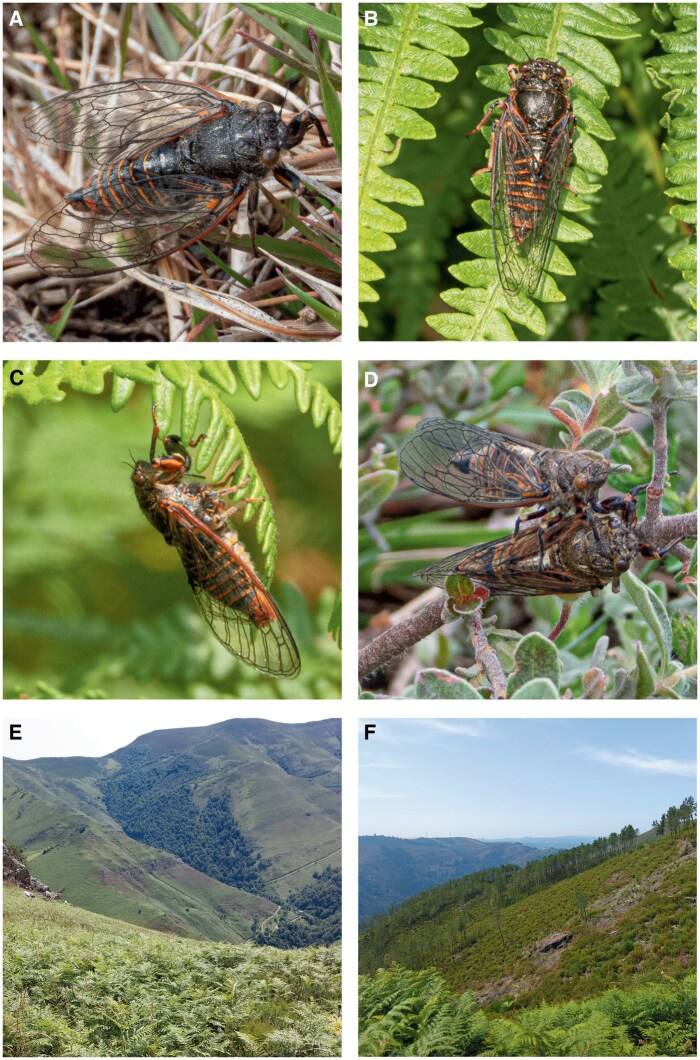
Alive specimens of *Cicadetta* sp. in different plants (A–D). Habitats of *Cicadetta* sp. in (E) pyrenean oak *Quercus pyrenaica* showing a preference for sunny heathland scrub habitats with *Erica*, *Cytisus*, *Ulex*, *Helianthemum*, *Pteridium aquilinum*, and other shrub species in Pico Gurrafa (Lena) and (F) in pine plantations of *Pinus* spp. showing a preference for sunny heathland scrub habitats with *Erica*, *Cytisus*, *Helianthemum*, and other shrub species, Trasmonte (Grandas de Salime).

Asturian specimens did not fit the description of known *Cicadetta* and may correspond to a new species/subspecies. Thus, we have treated it here as *Cicadetta* sp.

*Distribution and abundance.* This species has been recorded with a punctual and scattered distribution in three areas of Asturias: southwestern, southern, and also in an eastern area (Fig. 2C), associated with environments of intermediate thermal conditions, being scarce in the localities where it has been detected. A list of all records with more precise data can be found in Supplementary Material S2.

*Habitat.* The species has been found mostly in habitats with *Quercus pyrenaica* or *Quercus robur* woodland, typically on south-facing slopes, and also in sessile oak (*Quercus petraea* (Matt.) Liebl) (Fig. 6E and F). In most areas, *Cicadetta* occurs in small groves or isolated trees. These areas have shrubland vegetation, including species like *Helianthemum nummularium* (L.) Mill., three-toothed Broom (*Genista tridentata* L.), heath (*Erica arborea* L., *E. australis* L., and *E. cinerea* L.), and furze (*U. europaeus* L., *Ulex gallii* Planch.), brooms (*Cytisus cantabricus* (Willk.) Rchb.f. & Beck.), sage-leaved rock-rose (*Cistus salviifolius* L.), and a great abundance of eagle fern (*Pteridium aquilinum* (L.) Kuhn) (Fig. 6E and F). It was also found close to pine plantations of the Monterey pine (*Pinus radiata* D.Don) and *P. sylvestris*. Most of the areas of occupancy have a high recurrence of forest fires. Its altitudinal range spans from 300 to 1600 m a.s.l.

*Ecology and acoustic behavior.* In most of the locations visited, males of this species were heard singing, preferably perched in eagle ferns, *Pteridium aquilinum*. However, there were a few cases of males singing from the top of Pyrenean oaks, *Quercus pyrenaica*, and shrubs like *Helianthemum nummularium*. This taxon can sing at temperatures as low as 18 °C (on sunny days), showing more tolerance to the Atlantic climate as compared to its more thermophilous relatives. Males tend to stop singing below 17 °C. The species can be found from June to the end of July. The calling song is represented by high-pitch buzzes (12 to 17 kHz) with a total duration of 1.5 to 2.5 s (Fig. 3D). Each buzz is intercalated by a brief chirp and followed by a long pause of 1 to 2 s. This acoustic pattern resembles that of *Cicadetta brevipennis and C. petryi;* however, the song of the males recorded in Asturias does not fully match the acoustic descriptions of any *Cicadetta* taxon currently known in Europe.

**Fig. 7.**
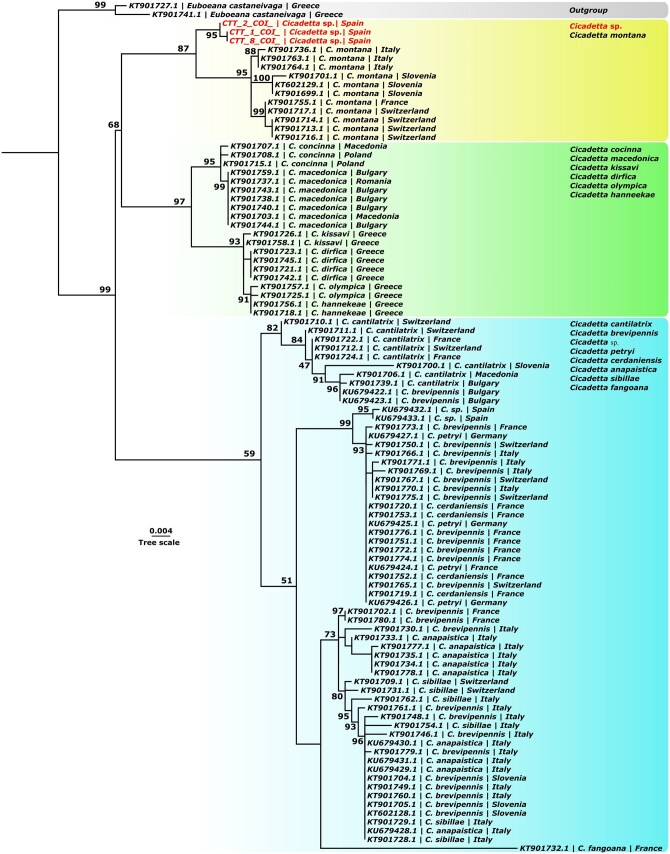
Maximum likelihood phylogeny inferred using the COI-3P dataset in IQ-TREE for *Cicadetta* sp. Ultrafast bootstrap values are provided at relevant nodes. GenBank accession numbers for sequences retrieved from GenBank are listed adjacent to each scientific name. New sequences from this study are highlighted in red.

**Fig. 8.**
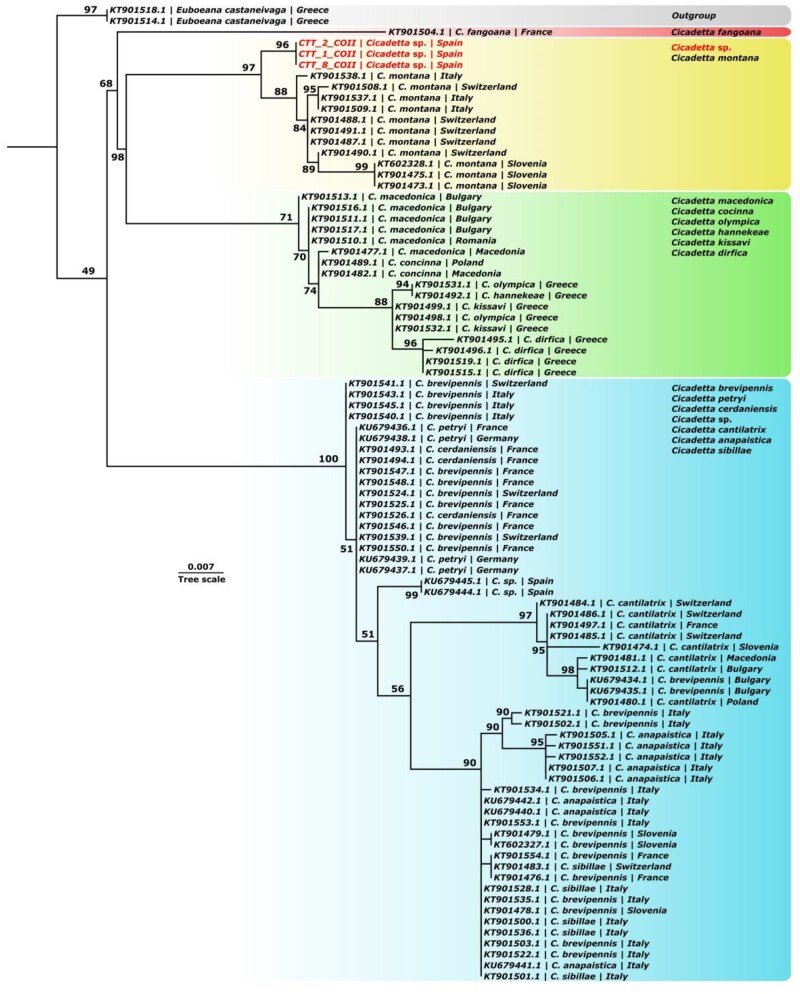
Maximum likelihood phylogeny inferred using the COII dataset in IQ-TREE for *Cicadetta* sp. Ultrafast bootstrap values are provided at relevant nodes. GenBank accession numbers for sequences retrieved from GenBank are listed adjacent to each scientific name. New sequences from this study are highlighted in red.

*Molecular identification.* A list of accession numbers of the newly generated sequences is available in Supplementary Appendix S1. The phylogenetic analysis (Figs 7 and 8) revealed a similar topology and clades previously reported by Hertach et al. (2016). Samples assigned to *Cicadetta* sp. based on morphology formed a well-supported group with a high bootstrap support in the two genes studied (ie 95% COI-3P; 96% COII) and located within a clade composed of *C. montana* (Scopoli, 1772) and *Cicadetta* sp. The COI-3P gene divergence (Fig. 7) ranges from 1.65% with *C. montana* to 5.85% with *C. brevipennis* Fieber, 1876. Among the compared taxa, *C. montana* is the species genetically closest to *Cicadetta* sp. Sequences for the COI-5P gene are not available in databases to make a proper comparison. Regarding the sequence of COII (Fig. 8) for *Cicadetta* sp. and the other congeners, it ranged between 5.22% with *C. anapaistica*  Hertach 2011 and 1.01% with *C. montana*, being this last one the genetically closest species to *Cicadetta* sp.

### Cicada Identification

**Fig. 9.**
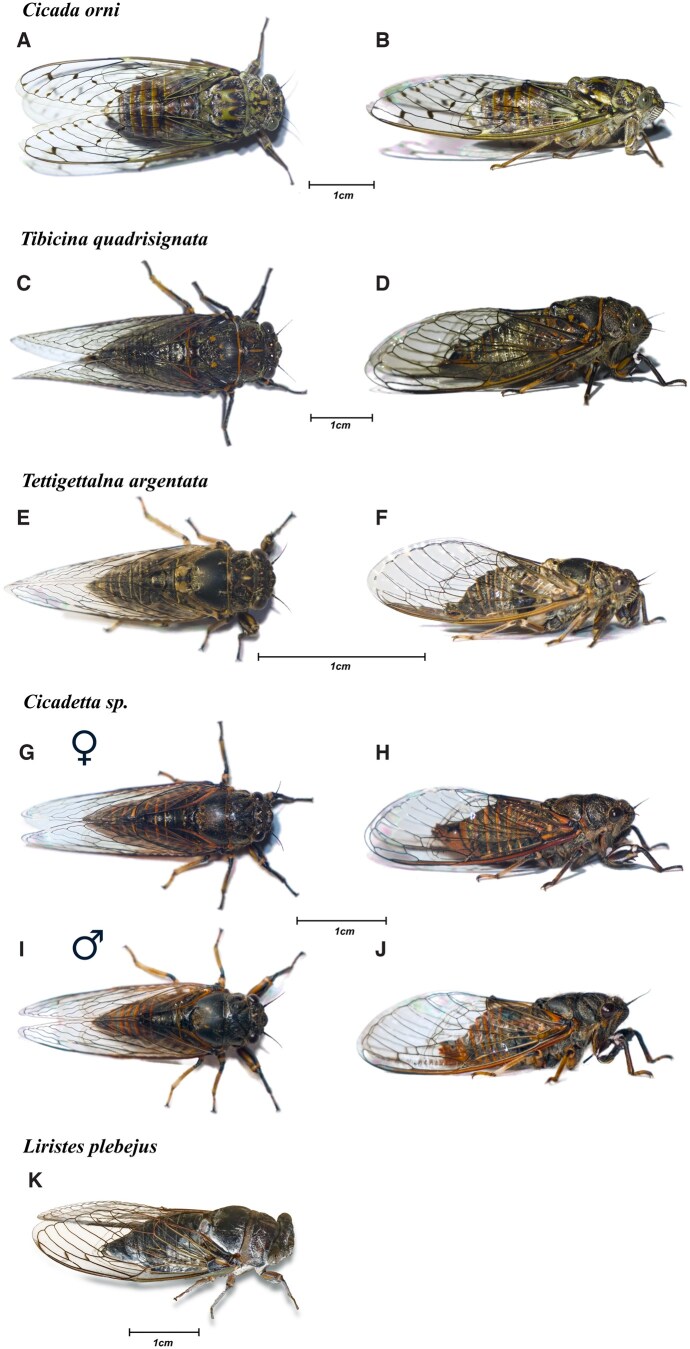
Dorsal and lateral photographs of specimens of cicada species collected in Asturias. *Cicada orni*, male (A, B); *Tibicina quadrisignata*, male (C, D); *Tettigettalna argentata*, male (E, F) and *Cicadetta* sp. female (G, H) and male (I, J). Additional photo of *Lyristes plebejus* from Cantabria (K).

To improve future cicada identification in Asturias, identification keys are provided in Supplementary Material S3, with references to sounds (Fig. 3) and morphology (Fig. 9). More information can be seen in Sannier and Sannier (2023).

### Potential Distribution Modeling

The models generated using the different algorithms achieved AUC values ranging from 0.99 to 1.00 and TSS values from 0.98 to 1.00 for *C. orni*, AUC values between 0.99 and 1.00, and TSS values between 0.95 and 0.99 for *Ti. quadrisignata*, and AUC values ranging from 0.97 to 0.99 with TSS values between 0.85 and 0.96 for *Te. argentata*. All these values indicate an excellent fit of the models produced by the different algorithms to the areas of occurrence of these species. In the case of *Cicadetta* sp., AUC values ranged from 0.79 to 1.00 and TSS values from 0.475 to 1.00, indicating a good performance of the algorithms used (Supplementary Material S4).

The variables with the greatest influence on the construction of the models for cicadas in Asturias were primarily those related to temperature (Supplementary Material S5). In contrast, precipitation of the driest month (Bio17) and slope had little relevance across all species and models (Supplementary Material S5). For *Cicadetta* sp. and *Te. argentata*, the most influential factor was the mean diurnal range (Bio2), with higher values of this variable corresponding to areas of greater suitability (Supplementary Material S5). In the case of *Ti. quadrisignata*, the maximum temperature of the warmest month (Bio5) was the key variable (Supplementary Material S5). For *C. orni*, three variables proved important: isothermality (Bio3), annual precipitation (Bio12), and, most notably, the maximum temperature of the warmest month (Bio5) (Supplementary Material S5).

**Fig. 10.**
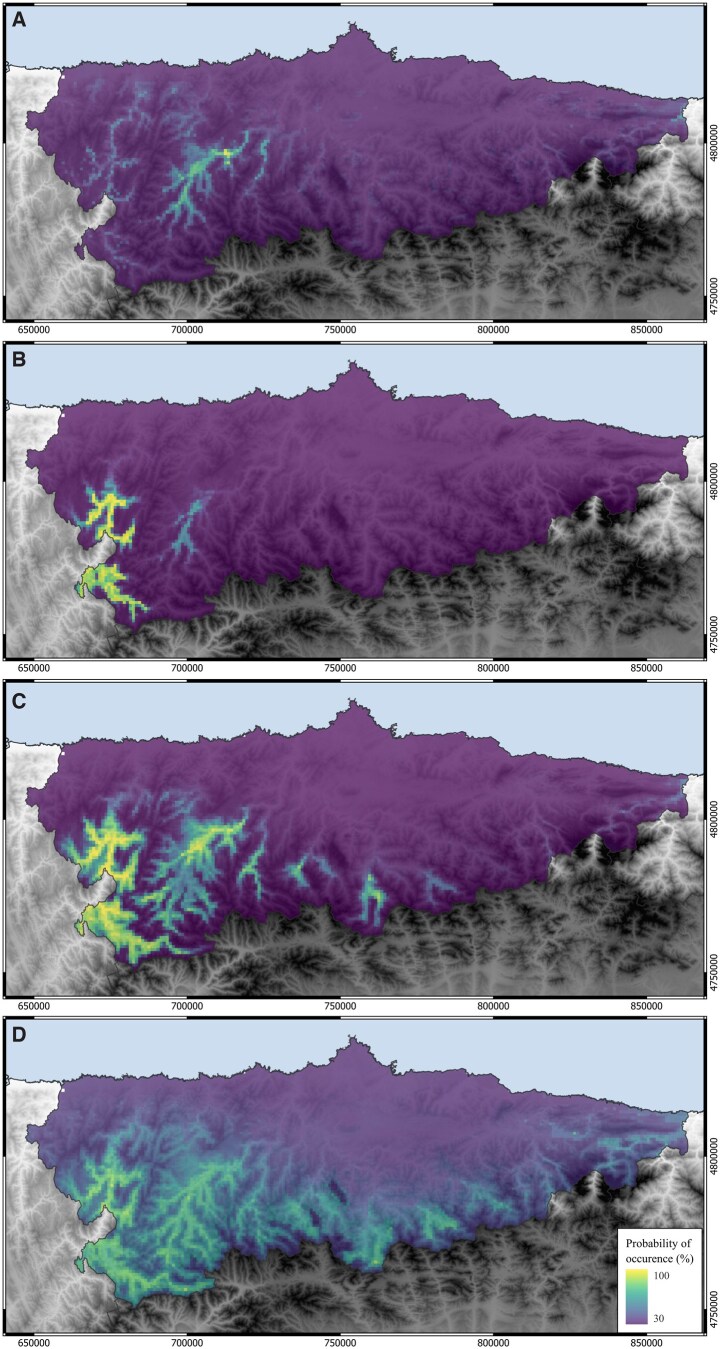
Potential distribution of Cicadidae in Asturias (A) *Cicada orni*, (B) *Tibicina quadrisignata*, (C) *Tettigettalna argentata*, and (D) *Cicadetta* sp.

Summarizing, three cicada species exhibit their areas of highest suitability in the warm river basins of Asturias: (1) *C. orni* shows high suitability only in the middle basin of the Narcea River (Fig. 10A) with intermediate values in the lower Deba and Pigüeña rivers; (2) *Ti. quadrisignata* is restricted to the warmest valleys (Navia-Oro and Ibias-Aviouga rivers) (Fig. 10B); and (3) *Te. argentata* across all thermal valleys (Navia-Oro, Ibias-Aviouga, and Narcea-Coto rivers), as well as the Lena-Pajares rivers (Fig. 10C). By contrast, *Cicadetta* sp. displays extensive areas of suitability in Asturias, associated with environments of intermediate thermal conditions (Fig. 10D).

## Discussion

The present study represents a significant advance in the knowledge of cicadas in the northern Iberian Peninsula and southwestern Europe, a region where these insects have received limited scientific attention due to the widespread assumption that temperate Atlantic environments are generally unsuitable for their persistence. Our results demonstrate that at least four cicada species belonging to different subfamilies are present in Asturias, although their occurrence is largely restricted to inland and mountainous areas that function as thermally favorable microclimatic (and sub-Mediterranean) refuges within a predominantly temperate oceanic landscape. The habitats occupied by the recorded species are broadly comparable to those used in other Iberian and European regions, being mainly associated with xerophilous shrublands and open, thermophilous woodland formations (Quartau et al. 2004, Puissant and Sueur 2010, Sanborn et al. 2011), supporting the interpretation of northern Iberia as a biogeographical transition zone. Across species, differences in distribution and abundance appear to reflect a clear gradient of climatic tolerance, with more thermophilous taxa (*C. orni* and *Ti. quadrisignata*; Quartau et al. 2000, 2004) restricted to warm valleys and south-facing and open slopes, and more temperate-adapted species (*Te. argentata* and *Cicadetta* sp.; Quartau et al. 2004, Pons et al. 2021) occurring over wider altitudinal ranges. Behavioral traits such as selection of perching height and vegetation strata for singing may act as thermoregulatory strategies under Atlantic conditions (*C. orni* and *T. quadrisignata* used higher perching spots on trees while *Te. argentata* and *Cicadetta* sp. used lower vegetation strata). Host plant use appears largely opportunistic, driven more by local vegetation structure and solar exposure than by strict plant specialization. Altogether, these results suggest that local microclimatic conditions may be more important than regional climate in determining the presence of cicada species. Furthermore, the potential occurrence of additional species in Asturias cannot be excluded. For example, *Lyristes plebejus* (Fig. 9K) has been confirmed for the first time in neighboring Cantabria (Deva river basin, near Potes; see Supplementary Material S3), only ∼10 km from Asturias in continuous suitable habitat (see Simões and Quartau 2013). Although not yet confirmed in Asturias, its presence in adjacent areas suggests a high likelihood of future detection (see Supplementary Material S2, and Figs 3E and 9K, for comparison).

In this study, we provide the first confirmed report of *C. orni* in Asturias. This species is widespread throughout southern Europe, where it occupies a broad range of woody habitats and host plants, typically associated with warm climates and closed shrubland or woodland formations (Quartau et al. 2004, Pinto-Juma et al. 2005). In Asturias, however, the species was detected only in the municipality of Tineo, although it has also been detected in Galicia, 1 km from the border with Asturias (Orois et al. in prep.) and in Cantabria, near Potes. The latter record belongs to the Deva river basin, where distribution models also support the possible presence of the species in its Asturian side. Its restricted distribution and low detectability in the region are likely linked to a combination of climatic constraints, demographic factors, and potential sampling bias. While *C. orni* may remain active from June to October in areas with hot summers (Quartau et al. 2000), our field data indicate a much shorter activity window in northern Iberia, limited from July to early August. This contraction is consistent with the relatively mild summer temperatures of Asturias, where mean maximum values rarely exceed 27.5 °C, potentially limiting both emergence synchrony and male singing activity (Quartau et al. 2000), and increasing the likelihood of missed detections during suboptimal survey conditions. Ecological niche models further support this interpretation, identifying only a few thermally favorable areas within the region as suitable for *C. orni*, largely restricted to warm inland valleys. Although additional populations may therefore exist and remain undetected, future surveys should prioritize thermophilous habitats during the hottest summer months. At the same time, the limited dispersal ability of *C. orni* and long larval development strongly constrain natural range expansion, as males are static singers and disperse only short distances from their emergence sites (Simões and Quartau 2007). Consequently, even under scenarios of climate warming that may promote earlier emergence and increased densities (Tsujimoto et al. 2024), the establishment of new populations in Asturias would likely be a slow process.

Regarding the *Tibicina* specimens detected in Asturias, morphological traits closely matched those of *T. quadrisignata*, a species that can be easily misidentified by *T. garricola* due to their remarkable morphological and acoustic similarity (Sueur and Aubin 2003, Quartau and Simões 2008). Historical records of *Tibicina* in Spain include several reports whose specific identification remains unconfirmed (eg Gómez-Menor Ortega 1957), highlighting taxonomic difficulties within the genus. Given the limitations of identification with morphology or bioacoustics alone, species determination in the present study was confirmed by genetic data, with COI sequences showing a high similarity (≈98%) to the reference sequence of *T. quadrisignata* from France (Sueur et al. 2007). The occurrence of *T. quadrisignata* in Asturias, confirmed in the present study, contributes to the fragmented and discontinuous distribution pattern reported across the Iberian Peninsula (Quartau et al. 2004, Pons et al. 2021, Puissant and Gurcel 2023) and southwestern Europe (Sueur and Puissant 2002, Quartau et al. 2004, Quartau and Simões 2008, Puissant and Gurcel 2023). This apparent fragmentation likely reflects not only ecological preferences for xeric and sub-xeric shrublands and open woodlands like pine forests (Sueur and Puissant 2002), but also persistent taxonomic uncertainty and identification constraints, which severely limit the reliability of citizen-science records and distribution databases, where most observations are currently reported only as *Tibicina* sp. Furthermore, phylogenetic and biogeographical knowledge of Iberian *Tibicina* remains incomplete, as the only available phylogeny of European Tibicininae is based on sparse and geographically biased sampling, lacking material from Spain and including only a few specimens from France and Portugal (Sueur et al. 2007). Moreover, acoustic divergence alone may not ensure reproductive isolation within *Tibicina*, with spatial segregation, habitat preferences, and phenological differences likely acting as additional isolating mechanisms (Sueur and Puissant 2002). Consequently, both the taxonomy and distribution of *T. quadrisignata* and related species in Iberia require reassessment through integrative approaches combining genetics, acoustics, ecology, and verified occurrence data. The scarcity of reliable records also constrains the accuracy of ecological niche models, underscoring the need for targeted fieldwork and a comprehensive revision of the genus in the Iberian Peninsula.

The small-sized Cicadettinae in Asturias are represented by the genera *Tettigettalna* and *Cicadetta*, with *Te. argentata* being by far the most widespread species recorded in the region. This species shows high ecological tolerance across the Iberian Peninsula, occupying a wide range of habitats from coastal lowlands to mountainous and even moderately disturbed areas, occurring often in sympatry with other cicada taxa (Quartau et al. 2004, Mendes et al. 2014, Nunes et al. 2014a, Mendes et al. 2023). In Asturias, morphological, acoustic, and genetic data consistently assign the studied populations to the northern clade of *Te. argentata* reported by Nunes et al. (2014b), a lineage that includes populations from the northwestern Iberian Peninsula and coastal Portugal and appears particularly tolerant of temperate climatic conditions. Phylogeographic analyses suggest that this northern clade originated through post-glacial colonization processes and represents the most recently diverged lineage, as all populations sampled north of the Pyrenees belong to this group (Costa et al. 2017, 2023). The wide distribution, habitat diversity, and relatively high local densities observed in Asturias further support *Te. argentata* as one of the most ecologically adaptable cicada species in the region. Individuals were observed singing at temperatures as low as 21 °C and frequently selected sunny, thermally favorable perching sites such as shrubs and roadside trees, stressing how thermoregulatory behavior likely facilitates persistence in temperate Atlantic environments. Additionally, ecological niche modeling predicts extensive areas of suitable habitat in southern Asturias, suggesting that the species may occupy a larger portion of the territory than currently detected.

Finally, the discovery of *Cicadetta* sp. populations in Asturias represents a remarkable novelty, as the genus has been rarely recorded in the Iberian Peninsula, with reports in the literature largely restricted to northeastern Spain (Hertach et al. 2016, Puissant and Gurcel 2018, Pons et al. 2021). The taxonomic identity of Spanish *Cicadetta* remains unresolved, as these populations have been classified under the *Cicadetta montana* complex, a group that has undergone extensive recent revision resulting in the recognition of multiple cryptic species and subspecies in recent years (Gogala et al. 2014, Hertach et al. 2015, 2016, Wade et al. 2015). Species within the *C. montana* complex are morphologically mostly indistinguishable but differ mainly in the acoustic parameters of male calling songs, which have been assumed as an effective premating isolation mechanism in these cicadas (Wade et al. 2015). The Asturian specimens clearly belong to the *C. montana* complex based on morphology, and their calling song fits within the *Cicadetta brevipennis* song group, characterized by a repeated binary pattern consisting of a long scheme followed by a short pause and a brief scheme (Hertach et al. 2016). However, none of the described taxa within this group fully matches the acoustic parameters recorded in Asturias, despite some similarity with *C. brevipennis and C. petryi.*

Genetic analyses further support the distinctiveness of the Asturian population, which forms a well-supported lineage clearly separated from other *Cicadetta* sequences available from Spain and France (Wade et al. 2015, Hertach et al. 2016). In particular, the Asturian *Cicadetta* shows marked genetic distance from its closest acoustic relatives, *C. brevipennis and C. petryi* (>5%, while 1.65% in the case of *Cicadetta montana s. str.*), indicating that it evolved as a separate and perhaps long-time isolated lineage. Notably, both its current and potential distributions indicate a better adaptation to cooler and more humid environments typical of Atlantic regions, a pattern that is also reflected in its ability to sing at lower temperatures and under conditions of reduced insolation. Taken together, the acoustic and genetic evidence strongly suggests that the *Cicadetta* population detected in Asturias represents a previously undescribed species. More detailed comparative morphological and acoustic data are required for a proper taxon description within the *Cicadetta montana* complex, which is beyond the scope of this study.

### Conservation Remarks

From a conservation perspective, our results indicate that climate change may have contrasting effects on cicada species in temperate Atlantic regions of Europe. Thermophilous taxa currently restricted to warm valleys and south-facing slopes may benefit from rising temperatures through longer activity periods (Moriyama and Numata 2019), increased detectability, and potential northward range expansion. In contrast, the *Cicadetta* population detected in Asturias appears better adapted to cooler and more humid conditions, showing activity at lower temperatures and under reduced insolation, which suggests a narrower climatic niche. Although its evolutionary and biogeographical history remains unresolved, this population could represent either a relict lineage or a taxon that has persisted locally under Atlantic conditions. In either case, further warming, together with increased drought stress and changes in vegetation structure, may negatively affect its habitat suitability and long-term persistence. These contrasting responses highlight the need to consider species-specific climatic tolerances and ecological requirements when assessing the conservation status of cicadas under ongoing environmental change. Fire regimes are also likely to interact strongly with these climatic effects. While moderate disturbance and fire-generated habitat mosaics may locally enhance insolation and favor thermophilous species, increasingly recurrent or high-severity fires may cause long term changes in vegetation structure, reduce host plant availability, and alter soil microclimatic conditions critical for cicada development, particularly given their long subterranean nymphal stages (Tobella et al. 2026). Moreover, post-fire management practices such as salvage logging may further exacerbate these impacts by removing structural refuges and increasing exposure to predators (Tobella et al. 2026). Notably, several of the cicada populations detected in this study occur in habitats subject to recurrent fire regimes in Asturias (Moreno et al. 2014), as well as in forest formations of timber interest, which may further threaten population persistence if disturbance intensity increases in the future. Taken together, these findings highlight the need for conservation and management strategies that account for species-specific climatic tolerances, fire regimes, and habitat heterogeneity in order to preserve cicada diversity, including potentially relict lineages, in temperate Atlantic landscapes under ongoing environmental change.

## Supplementary Material

ieag065_Supplementary_Data
