# Supplementary material for: Echoes from northern Iberia: distribution, ecology, genetics, and identification of Asturian cicadas (Hemiptera: Cicadidae)
Source: J Insect Sci. 2026 Jun 30;26(3):ieag065. doi: 10.1093/jisesa/ieag065 (PMC13387362; doi:10.1093/jisesa/ieag065)
Supplement: ieag065_Supplementary_Data [file ieag065_supplementary_data.zip › Supplementary_Material_3._Identification.docx]

**Supplementary Material 2.** Simplified dichotomous visual and acoustic keys for Asturian species (including *Lyristes plebejus* as a potential species) for non-experts. Figures are included in the text.

**Visual identification key**

The most reliable characters for easily distinguishing the species are overall body size and shape, colour markings in dorsal view, wing venation and male calling song (Sannier and Sannier 2023). More illustrations are available at Pons et al. (2021).

**1.** Robust cicadas of large body (length 30-50 mm) ………………………………………………………..……......…………………………...………...….……... **2.**

— Small cicadas (length < 30 mm) …………………..…………….……………………………………………………………..………………….. **4.**

**2.** Forewings with dark spots or darkened crossveins …………………………………………………………………………………………..……...….……………..**3.**

— Hialine forewings without any dark spot; dark blackish body with four conspicuous reddish–orange markings on the thorax (mesonotum); veins at the base of the wings show vivid orange colour (visible with wings open) ………………………………………………………..***Tibicina quadrisignata*** (Fig. 8c-d).

**3.** —Forewing shows 10 dark apical markings on the veins; overall grey-brown colour; the tip of the abdomen is white; there is an olive-green or beige midline marking near the head (pronotum midline) and an olive-green or light-brown dorsal W in the thorax (mesonotum) ………………… …………………………………………………………………………………………. ***Cicada orni*** (Fig. 8a-b).

— Hialine forewings with 2 darkened apical crossveins; very large, wide apart and protruding eyes; quite robust and hunchback dorsum (mesonotum); overall dark colour with some notes of light orange/beige …….………………………………..………………..…………… ***Lyristes plebejus*** (Fig. 8k).

**4.** Overall dark body with bright and vivid orange markings, clearly visible on the outer margins of the wings and on the abdomen rings. On the base of the wing, the first cubital nerve is inserted in the union of basal and radial nerves .…………………………...… ***Cicadetta* sp*.*** (Asturian populations) (Fig. 8g-j).

— Overall coloration duller and more subdued; yellowish orange markings weak or poorly marked on wings and abdomen. On the base of the wing, the first cubital nerve is directly inserted into the radial nerve………………………………..………………..………………….. ***Tettigettalna argentata*** (Fig. 8e-f).

**Acoustic identification key**

Sonogram are from Asturian species, including *L. plebejus* also as a potential species. In particular, *Tibicina* species are considered in two groups due to the difficulty of their differentiation in the field, and *Cicadetta* taxa are treated *sensu lato* until the taxonomic status of the Asturian population is resolved.

**1.** Song discontinuous, consisting of short (5-6 seconds) phrases, each one made up of a long whistle, gradually louder, followed by a short, more audible pulse at the end ……...….. ***Cicadetta*** spp (Fig. 9d).

— Song continuous, with more than 30 seconds of uninterrupted singing, usually much more ……... **2.**

**2.** Song uniform, with no breaks, a continuous high-pitched and deafening noise ……………………………………………………...…….…………….……***Tibicina quadrisignata*** (Fig. 9b).

— Song broken into short pulses, similar to the sound of a machine-gun ……………………….……… **3.**

**3.** Song made up of disorderly and chaotic pulses, often a sequence of very fast pulses followed by a few seconds of continuous sound …………………………...……………..…. ***Lyristes plebejus*** (Fig. 9e).

— Song comprising rhythmic and constant pulses, separated by breaks of homogenous duration … **4.**

**4.** Pulses high-pitched and fast (≈10 pulses/second), with short breaks every 3-4 seconds of song ……………………………………………………………………………… ***Tettigettalna argentata*** (Fig. 9c).

— Pulses lower-pitched and slower (≈7 pulses/second), reminiscent of a scratching record, very loud and often deafening …………………………………………………………………… ***Cicada orni*** (Fig. 9a).
