# Supplementary material for: Echoes from northern Iberia: distribution, ecology, genetics, and identification of Asturian cicadas (Hemiptera: Cicadidae)
Source: J Insect Sci. 2026 Jun 30;26(3):ieag065. doi: 10.1093/jisesa/ieag065 (PMC13387362; doi:10.1093/jisesa/ieag065)
Supplement: ieag065_Supplementary_Data [file ieag065_supplementary_data.zip › Titles_of_Supplementary.docx]

**Supplementary Material 1.** Bioclimatic variables used in the spatial distribution models. Variables marked with an asterisk were used in the final models

**Supplementary Material 2.** Cicadidae records from the study area of the considered species.

**Supplementary Material 3.** Simplified dichotomous keys for Asturian species for non-experts.

**Supplementary Material 4.** Importance of each variable used in the models for each Cicadidae species in Asturias a) *Cicada orni*, b) *Tibicina quadrisignata*, c) *Tettigettalna argentata* and d) *Cicadetta* sp.

**Supplementary Material 5.** Importance of each variable used in the models for each Cicadidae species in Asturias a) *Cicada orni*, b) *Tibicina quadrisignata*, c) *Tettigettalna argentata* and d) *Cicadetta* sp.
