# Supplementary material for: Echoes from northern Iberia: distribution, ecology, genetics, and identification of Asturian cicadas (Hemiptera: Cicadidae)
Source: J Insect Sci. 2026 Jun 30;26(3):ieag065. doi: 10.1093/jisesa/ieag065 (PMC13387362; doi:10.1093/jisesa/ieag065)
Supplement: ieag065_Supplementary_Data [file ieag065_supplementary_data.zip › Appendix_1_&_2.docx]

# **Appendix 1**

Geographic data and GenBank accession numbers for the Asturian (all in Spain) cicadas specimens sequenced in this study.

| **Species** | **Code** | **Col. Date** | **Location** | **COI-5P** | **COI-3P** | **COII** | **Coord.** |
| --- | --- | --- | --- | --- | --- | --- | --- |
| *Cicadetta* sp. | CCT-1 | 4-VII-2023 | La Cava, Lena, Asturias | PX526119 | PX526116 | PZ016759 | 43,09795 -5,72088 |
| *Cicadetta* sp. | CTT-2 | 4-VII-2023 | La Cava, Lena, Asturias | PX526120 | PX526117 | PZ016760 | 43,08451 -5,74021 |
| *Cicadetta* sp. | CTT-8 | 3-VII-2023 | La Gurrafa, Lena, Asturias | PX526121 | PX526118 | PZ016761 | 43,08397 -5,73337 |
| *Tibicina quadrisignata* | TIB-1 | 16-VII-2025 | Zoío-Seroiro, Ibias, Asturias | PX467324 | PX467325 | – | 43,05724 -6,82575 |
| *Tettigettalna argentata* | TTT-1 | 23-VI-2023 | Encinar Piridillu, Lena, Asturias | PX467329 | – | – | 43,13592 -5,81402 |
| *Cicada orni* | ORN-2 | 16-VII-2025 | Soto La Barca, Tineo, Asturias, | PX467326 | – | – | 43,29517 -6,37569 |

# **Appendix 2**

List of species listed for Northern regions of the Iberian Peninsula according to checklists available in the literature and references therein: (×) and (–) denote confirmed presence or no presence, respectively. There are no mentions in the literature referring specifically to the northern regions of Galicia, Asturias, Cantabria, País Vasco, Navarra or La Rioja. Information presented was retrieved from the following checklists/studies: Aguin-Pombo et al. 2007; Hertach et al. 2016 and Puissant & Gurcel, 2018 for Aragón, Pons et al. 2012 for Catalonia, and Sueur et al. 2004 for the northern half of Portugal. The last column infers the putative presence of these species in Asturias based on their known distribution range and habitat preferences.

| **Subfamily** | **Species** | **Aragon** | **Catalonia** | **N Portugal** | **Habitat type** | **Asturias?** |
| --- | --- | --- | --- | --- | --- | --- |
| **Cicadinae** | *Cicada barbara* (Stål, 1866) | – | × (Accidental) | × | Shrubland, woodland | Unlikely |
|  | *Cicada orni* Linnaeus, 1758 | × | × | × | Woodland | Present |
|  | *Cicadatra atra* (Olivier, 1790) | – | × | – | – | Possible |
|  | *Lyristes plebejus* (Scopoli, 1763) | × | × | × | Shrubland, woodland | Likely |
| **Tibicininae** | *Tibicina garricola* Boulard, 1983 | – | × | × | Shrubland, woodland | Possible |
|  | *Tibicina haematodes* (Scopoli, 1763) | × | × | – | – | Likely |
|  | *Tibicina quadrisignata* (Hagen, 1855) | × | × | × | Shrubland, woodland | Present |
|  | *Tibicina tomentosa* (Olivier, 1790) | × | × | – | Moorland, shrubland | Unlikely |
| **Cicadettinae** | *Cicadetta petryi* Schumacher, 1924 | × | × | – | – | Possible |
|  | *Cicadetta cerdaniensis* Puissant & Boulard, 2000 | × | – | – | – | Possible |
|  | *Euryphara dubia* (Rambur, 1840) | × | × | – | Grassland | Unlikely |
|  | *Hilaphura varipes* (Waltl, 1837) | × | × | – | Grassland, shrubland | Unlikely |
|  | *Oligoglena tibialis* (Panzer, 1798) | ×? | – | – | – | Unlikely |
|  | *Tettigettalna argentata* (Olivier, 1790) | × | × | × | Grassland, moorland, shrubland, woodland | Present |
|  | *Tettigettalna estrellae* (Boulard, 1982) | – | – | × | Shrubland, woodland | Unlikely |
|  | *Tympanistalna gastrica* (Stål, 1854) | – | – | × | Shrubland | Unlikely |
