# Supplementary material for: Echoes from northern Iberia: distribution, ecology, genetics, and identification of Asturian cicadas (Hemiptera: Cicadidae)
Source: J Insect Sci. 2026 Jun 30;26(3):ieag065. doi: 10.1093/jisesa/ieag065 (PMC13387362; doi:10.1093/jisesa/ieag065)
Supplement: ieag065_Supplementary_Data [file ieag065_supplementary_data.zip › Supplementary Material 1.docx]

**Supplementary Material 1.** Bioclimatic variables used in the spatial distribution models. Variables marked with an asterisk were used in the final models.

Bio1: annual mean temperature.

*Bio2: mean diurnal range (mean of monthly (max temp - min temp)).

*Bio3: isothermality (Bio2/Bio7) (×100).

Bio4: temperature seasonality (standard deviation ×100).

*Bio5: max temperature of warmest month.

Bio6: min temperature of coldest month.

Bio7: temperature annual range (Bio5-Bio6).

Bio8: mean temperature of wettest quarter.

Bio9: mean temperature of driest quarter.

Bio10: mean temperature of warmest quarter.

Bio11: mean temperature of coldest quarter.

*Bio12: annual precipitation.

Bio13: precipitation of wettest month.

Bio14: precipitation of driest month.

Bio15: precipitation seasonality (coefficient of variation).

Bio16: precipitation of wettest quarter.

*Bio17: precipitation of driest quarter.

Bio18: precipitation of warmest quarter.

Bio19: precipitation of coldest quarter.

Elev: elevation.

*Slope: terrain slope.

Irreg: terrain irregularity.

TRI: terrain ruggedness index.
