# Supplementary figures and images for: Echoes from northern Iberia: distribution, ecology, genetics, and identification of Asturian cicadas (Hemiptera: Cicadidae)
Source: J Insect Sci. 2026 Jun 30;26(3):ieag065. doi: 10.1093/jisesa/ieag065 (PMC13387362; doi:10.1093/jisesa/ieag065)

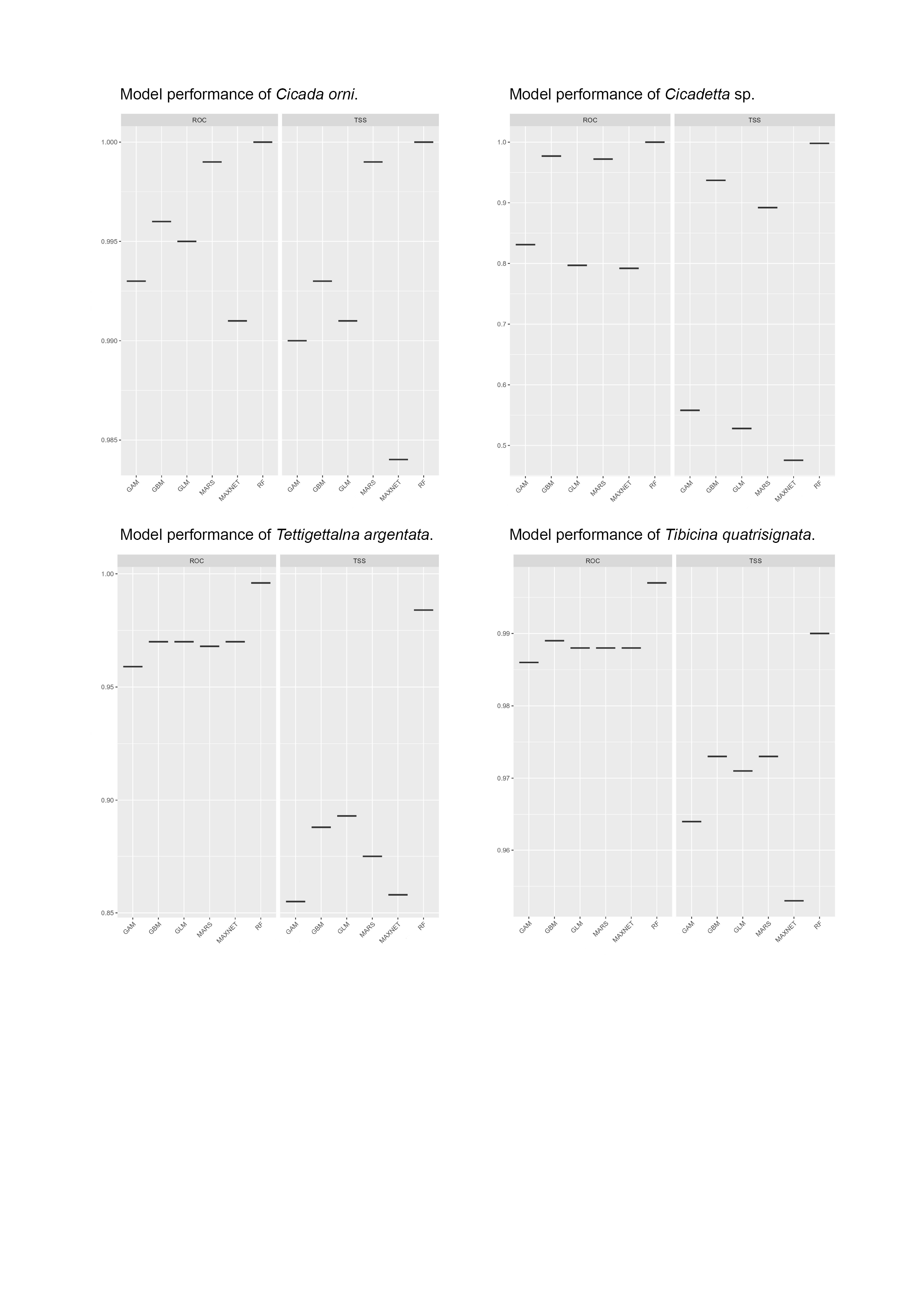

Supplement: ieag065_Supplementary_Data [file ieag065_supplementary_data.zip › Supplementary Material 4.jpg]

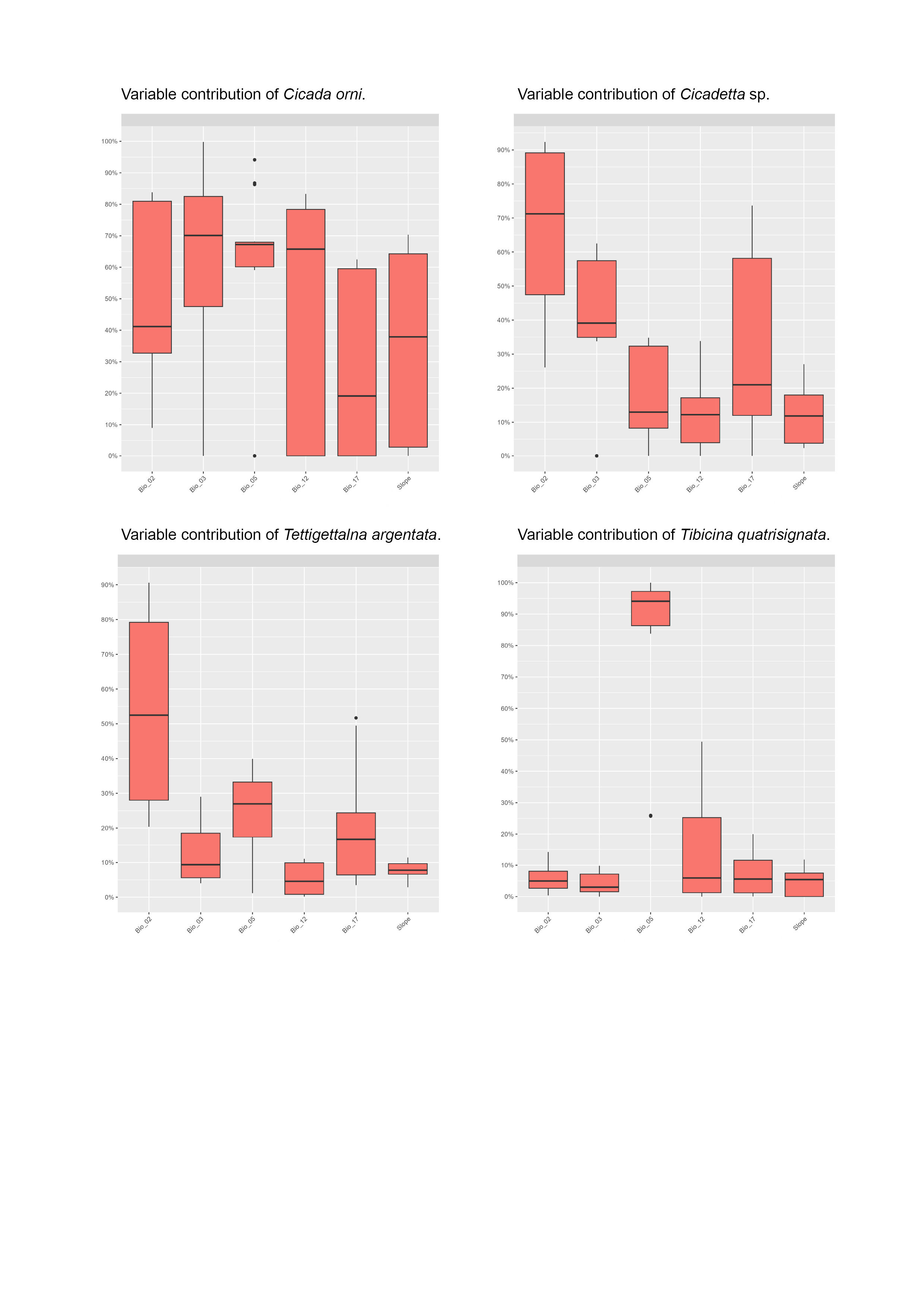

Supplement: ieag065_Supplementary_Data [file ieag065_supplementary_data.zip › Supplementary Material 5.jpg]
